# Economic evaluation of agomelatine relative to other antidepressants for treatment of major depressive disorders in Greece

**DOI:** 10.1186/1472-6963-13-173

**Published:** 2013-05-10

**Authors:** Nikos Maniadakis, Georgia Kourlaba, Theodoros Mougiakos, Ioannis Chatzimanolis, Linus Jonsson

**Affiliations:** 1 Department of Health Services Organization & Management, National School of Public Health, 196 Alexandras Avenue, Athens 11521, Greece; 2 Psychiatric clinic, 414 Military Hospital, Athens, Greece; 3 Psychiatry Department, National and Kapodistrian University of Athens, Eginition Hospital, Athens, Greece; 4 Optuminsight, Stockholm, Sweden

**Keywords:** Depression, Cost-effectiveness, Cost-utility, Agomelatine, Greece

## Abstract

**Background:**

Major depressive disorder (MDD) constitutes an important public health problem, as it is highly prevalent in the industrialized world and it is associated with substantial economic consequences for patients, health care providers, insurance and social security organizations and employers. To conduct an economic evaluation comparing agomelatine with other commonly used alternatives for treating patients with major depressive disorder (MDD) in Greece.

**Methods:**

An existing international Markov model designed to evaluate the cost-effectiveness of agomelatine was adapted to the Greek setting. It reflects six different health states, in which patients may move on a monthly basis. The analysis was undertaken from a societal perspective. Transition probabilities, utilities and costs assigned to each health state were extracted from the published literature, government sources and expert opinion. Data reflects the year 2012 and was discounted using a rate of 3.5%. Probabilistic analysis was undertaken to deal with uncertainty.

**Results:**

Base case analyses revealed that agomelatine is a dominant therapy for MDD relative to escitalopram, fluoxetine and sertraline, and it appeared to be cost-effective compared to venlafaxine (ICER: €547/QALY). Agomelatine remained a dominant treatment against generic sertraline and fluoxetine, and it appeared to be a cost-effective alternative compared to generic venlafaxine and escitalopram (ICER: €1,446/QALY and €3,303/QALY, respectively). Excluding the indirect cost from the analysis, agomelatine remained a cost-effective alternative over all comparators. In the probabilistic sensitivity analysis agomelatine was dominant in 44.5%, 89.6%, 70.6% and 84.6% of simulated samples against branded venlafaxine, escitalopram, fluoxetine and sertraline, respectively.

**Conclusion:**

The present evaluation indicates that agomelatine is either a dominant or a cost-effective alternative relative to branded or generic alternatives, in Greece.

## Background

Major depressive disorder (MDD) constitutes an important public health problem, as it is highly prevalent in the industrialized world and it is associated with substantial economic consequences for patients, health care providers, insurance and social security organizations and employers [1]. The burden of depression is significant in terms of direct treatment cost, social and intangible cost, missed working days, lower productivity, and decreased quality of life. In Europe, evidence coming from several different studies, indicates that the prevalence of major depression ranges between 3.1% and 10.1% [2]. In this light, the total annual burden of depression in Europe has been estimated at €118 billion in 2004, making it the most costly of all brain disorders, accounting for nearly 33% of their overall cost. The direct health care cost accounts for 36% of the total burden of MDD and this is mainly due to outpatient care (€22 billion), drug utilisation (€9 billion) and hospitalizations (€10 billion). Indirect costs due to morbidity and mortality associated with depression account for 64% of its total burden [1].

Untreated, severe depression is related to increased risk of suicide, psychiatric hospitalizations, and to substantial productivity loss as a result of long-term absenteeism from work [3,4]. Not surprisingly, full remission results in significantly lower costs and higher quality of life compared to no or only partial response [5]. These highlight the immense need for an effective management of depression both for health as well as for economic reasons. The treatment of MDD requires complex multimodal therapy which is dependent upon the state of the illness. Specifically, the treatment of depression includes pharmacotherapy, psychotherapy and other therapies [6]. Whereas pharmacotherapy is not always required for less severe forms of depression, severe depression usually requires the use of antidepressants, which nowadays play an important role in the effective management of depression [6]. There are many different classes of antidepressants, including: selective serotonin reuptake inhibitors (SSRIs), selective norepinephrine reuptake inhibitors (SNRIs), tricyclic antidepressants (TCAs), non-TCAs, and monoamine oxidase inhibitors (MAOIs) [7-10].

Agomelatine represents the first melatonergic agonist (MT_1_ and MT_2_ receptors) and 5-HT_2C_ antagonist [11,12] antidepressant and received a marketing authorization by the European Medicines Agency in 2009. A recently published review aiming to evaluate the safety and efficacy of agomelatine for the treatment of depression, showed that agomelatine was safe and its overall tolerability profile was superior to selective serotonin reuptake inhibitors (SSRIs) or selective serotonin and norepinephrine reuptake inhibitors (SNRIs) [12]. Moreover, a meta-analysis aiming to evaluate the efficacy of agomelatine in MDD revealed that agomelatine was superior to placebo and a number of selected anti-depressants [13].

In the recent climate of financial constraints it is particularly important to evaluate the effectiveness of new therapies (i.e. life years or quality-adjusted life-years (QALYs) gained) in relation to their long-term costs relative to existing ones, in order to determine the most efficient care that can be delivered to patients from existing resources. Therefore, cost-effectiveness studies that value medications and other technologies not only on the basis of clinical efficacy, but also incorporate considerations of tolerability, safety, and estimates of resource consumption should be conducted. This type of analysis reveals whether the new treatments provide “good value for money” and are worth their investment. Cost-effectiveness analysis in depression generally requires modelling, as all the required data are rarely available from a single study over the relevant timeframe [14]. Previous economic evaluations of treatments for depression have been focused mainly on newer generations of anti-depressant drugs (SSRI/SNRI) [15]. However, no published study comparing agomelatine with SSRIs and SNRIs in terms of cost-effectiveness is available, to our knowledge. Therefore, the purpose of the present study was to conduct an economic evaluation comparing agomelatine (Valdoxan®) with its most common alternatives in daily clinical practice for treating patients with MDD in Greece.

## Methods

In the present study, an existing Markov model evaluating the 2-year cost-effectiveness of agomelatine relative to various other antidepressants in the management of MDD patients was adapted to the Greek health care setting. This specific model has been submitted to European Health Technology Assessment agencies (http://www.tlv.se/Upload/Beslut_2010/bes101028-valdoxan.pdf) and it was developed based on validated and published methodologies [16,17]. The comparators (i.e. venlafaxine, escitalopram, fluoxetine, and sertraline) were selected based on their market shares in Greece. The analysis was conducted from a Greek societal perspective. Costs and outcomes that occur beyond one year were discounted at a 3.5% annual rate which is the standard practice in these studies in Greece [18].

### Model structure

**Figure 1:**
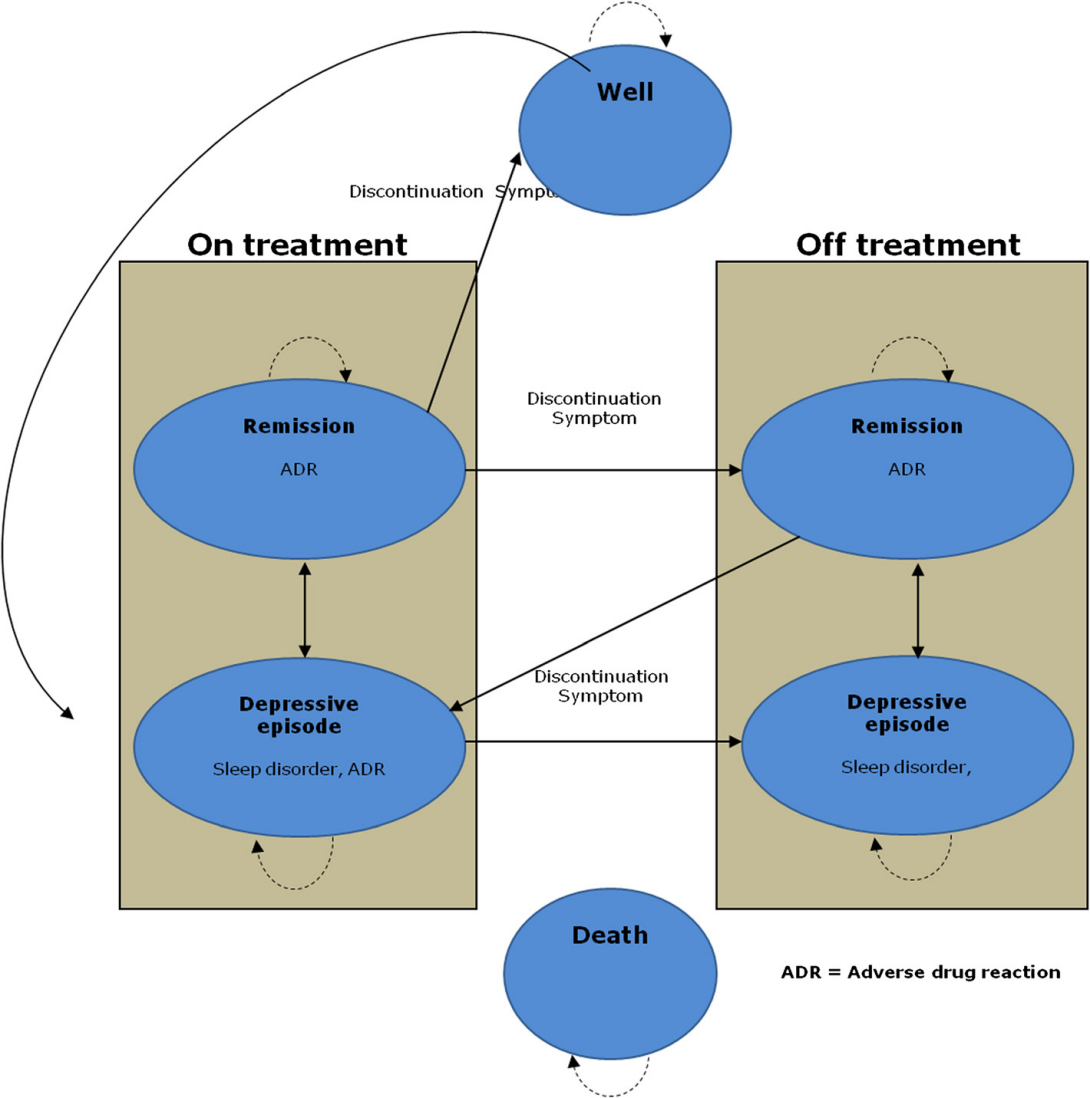
Structure of the model.

The model, which is outlined in Figure 1, consists of six health states: healthy, depressive episode on treatment, remission on treatment, depressive episode off treatment, remission off treatment and death. The cycle length of the model was set to 1 month. In particular, a patient with a mean age of 45 years enters the model in the “depressive episode” state and he experiences a probability of remitting and thus moving to the state “remission”. Once in remission, the patient may suffer a relapse and enter a new depressive episode, or the patient may move on to the healthy state (after spending six months in the state of remission [19]). In the healthy state, there is a risk of suffering a recurrence and thus enter a new depressive episode. In the case of relapse, the patient always returns to the original treatment. In each state there is an associated mortality risk, which is independent of the state, except for the case of a depressive episode, which is adjusted with a multiplication factor to reflect the increased risk of suicide. During a depressive episode the patient may suffer an adverse drug event or sleep disorder. Assuming that the sleep disorder is related to the depression itself, during remission the patient may only suffer an adverse drug event. Such events occur with a fixed probability and are associated with a utility reduction and a cost increase.

While on the original treatment during either an episode or remission, the patient may discontinue treatment. The risk of discontinuation is treatment-specific and by definition is set to zero for placebo. In the case of discontinuation, the patient incurs a treatment specific risk of suffering from discontinuation symptoms. The risk of discontinuation symptoms also alters as the patient moves to the healthy state. Antidepressant medication is assumed to be administered during both a depressive episode and remission. Beginning in the second period of a depressive episode and continuing through remission, the cost of medication is increased to reflect the dose adjustment observed in the clinical trials.

### Model inputs

The results of the model are driven by many different input parameters, including the probabilities of death, remission, relapse, recurrence, discontinuation and adverse event for each therapy option, as well as the corresponding utility values and cost associated with each health state. Moreover, the treatment doses utilized and the proportion of patients receiving double dose are also significant model input parameters. The corresponding data was derived from the published literature, government sources, and expert opinion (T.M and I.C) and is used as outlined below.

#### Doses and dose increase

Treatment doses, as well as dose increases were taken from clinical trials in order to correlate doses with clinical efficacy. Regarding Agomelatine, the standard dose was used for the initiation of the treatment (25 mg/day). After a period of one cycle the percentage of patients receiving double dose of Agomelatine was derived through the meta-analysis of data from the head-to-head clinical trials [20-23] (Table 1). Treatment doses for venlafaxine, fluoxetine and sertraline, as well as dose increase were taken from the corresponding Agomelatine head-to-head clinical trials [20-22]. There were no available data for escitalopram regarding the dose used in the clinical trial and the percentage of patients that received double dose. Thus a 20 mg/day dose was assumed, which seems to be in line with published literature [24].

**Table 1:** Model input parameters

**Parameters**	**Agomelatine**	**Venlafaxine**	**Fluoxetine**	**Sertraline**	**Escitalopram**
Dose	25 mg	75 mg	20 mg	50 mg	20 mg
Percentage of patients receiving double dose	22.1%	11.8%	23.0%	24.5%	0.0%
Remission	0.323 [20-22]	0.323*	0.284 [22]	0.289 [21]	0.323*
Recurrence	20% [25]	20% [25]	20% [25]	20% [25]	20% [25]
Suicide risk	20.35 [26]	20.35 [26]	20.35 [26]	20.35 [26]	20.35 [26]
Discontinuation	0.117 [20-23]	0.216 [20]	0.171 [22]	0.189 [21]	0.144 [23]
Discontinuation symptoms	0.00 [27]	0.20 [27]	0.00	0.00	0.07
Relapse	Survival function [28]	Survival function [28]	RR:0.513^§^	RR:0.633^§^	RR:0.531^§^
Constipation	0.027 [20-23]	0.042 [20]	0.011 [22]	0.006 [21]	0.006 [23]
Dyspepsia	0.020 [20-23]	0.024 [20]	0.008 [22]	0.013 [21]	0.025 [23]
Diarrhoea	0.040 [20-23]	0.018 [20]	0.027 [22]	0.057 [21]	0.069 [23]
Nausea	0.066 [20-23]	0.226 [20]	0.114 [22]	0.044 [21]	0.138 [23]
Somnolence	0.035 [20-23]	0.048 [20]	0.034 [22]	0.013 [21]	0.038 [23]
Headache	0.111 [20-23]	0.119 [20]	0.114 [22]	0.101 [21]	0.144 [23]
Sexual Dysfunction	0.005 [20-23]	0.018 [29]	0.004 [22]	0.019 [21]	0.013 [23]
Sleep Disorder	0.007 [20-23]	0.024 [20]	0.019 [22]	0.019 [21]	0.025 [23]

^*^assumed to be equal to agomelatine due to lack of relevant data.

^§^Meta-analysis vs. Placebo.

#### Remission rate

Clinical data from head-to-head clinical trials of agomelatine relative to venlafaxine [20], fluoxetine [22] and sertaline [21] was used to obtain a pooled estimate of the remission rate for agomelatine group. The head-to-head clinical trial of agomelatine versus escitalopram [23] provides heterogeneous information (higher than the average remission rates), not directly comparable to the other studies, because of a different study duration (i.e. 12, 8 and 6 weeks). Hence, this study was not incorporated into the pooled estimation of remission rate for agomelatine. In the model patients should spend 6 months on the remission state before they move to “well” state.

#### Discontinuation rate and discontinuation symptoms

Discontinuation rates for the comparison of agomelatine, venlafaxine, sertraline, fluoxetine and escitalopram were derived from head-to-head clinical trials comparing the therapies [20-23]. The frequency of discontinuation symptoms was set to zero for agomelatine because a randomized clinical trial conducted to examine the effect of an abrupt interruption of agomelatine [27] concluded that there was an absence of discontinuation symptoms in agomelatine-treated patients, whilst discontinuation symptoms were detected in paroxetine-treated patients. Another study aimed to evaluate discontinuation symptoms in depression and anxiety disorders showed no significant difference between paroxetine and venlafaxine [30]. Therefore, in the present analysis, the frequency of discontinuation symptoms in paroxetine-treated patients was applied to the venlafaxine arm [27]. The frequency of discontinuation symptoms was also set to zero for all other active comparators for which no data was available. The length of discontinuation symptoms was set to 1 week [27].

#### Relapse rate

For the comparison of agomelatine relative to venlafaxine or placebo, the probability of relapse was estimated on the basis of data obtained from a study designed as a relapse prevention study [28,31]. A Weibull survival curve was fitted (Additional file 1). Assuming equivalent anti-depressant effect on relapse of agomelatine and venlafaxine the same survival curve was used for both comparators in the model (Table 1). Relapse rates for the remaining active comparators were modeled through the relative risks (RR) of comparators versus placebo (Table 1). These RRs were derived from a meta-analysis of available placebo-controlled trials for each comparator involved in the present study, due to the absence of relevant data from head-to-head clinical trials.

#### Adverse drug reactions

Adverse drug reaction (ADR) rates for venlafaxine, sertraline, fluoxetine and escitalopram were obtained directly from agomelatine head-to-head clinical trials [20-23]. The corresponding rates for agomelatine were obtained from pooled analysis of these studies. The monthly frequencies of all adverse events as well as the studies used to extract this data are presented in Table 1.

#### Mortality

The probability of death was estimated on the basis of Greek mortality rates taken from the latest publication from the National Statistics Service (http://www.statistics.gr). To reflect the increased risk of patients with mental disorders associated to high suicide rates in such patients, general mortality was multiplied with a factor (20.35) to model the increased relative risk during depressive episodes [26].

#### Recurrence

Due to lack of published national data regarding the recurrence risk of depression, data published from Angst et al. was used in the model [25].

##### Utility values

Since local utility values for patients treated for depression are lacking, the values proposed by Sobocki et al. for each health state (i.e. healthy, remission, and depressive episode) were used in the present analysis [17,32]. Moreover, the utility decrements proposed by Sullivan et al. were used in the case of different adverse event reactions [33]. Regarding insomnia, Botteman et al. estimated that the utility reduction is 0.08095 [34], while Sullivan et al. estimated that this is 0.129 [33]. In the model, the lower estimate was used, thus ensuring a conservative approach. Finally, Montgomery et al. showed that nausea was a frequently occurring symptom following discontinuation from paroxetine [27]. A decision was therefore made to set the disutility due to discontinuation symptoms equal to that of nausea (Table 2).

**Table 2:** Utility values, utility decrease and costs assigned to health states and adverse drug reactions (ADR)

	**Utility values**	**Costs in €**
**Health State**	**Utility**	**Monthly Cost**
*Healthy*	0.86 [17]	0
*Remission*	0.81 [17]	Direct: 35^‡^
		Indirect: 173^†^
*Depressive Episode*	0.57 [17]	Direct: 190^‡^
		Indirect: 380^†^
**Adverse Drug Reaction**	**Utility Decrease**	Monthly cost*
*Constipation*	0.065 [33]	12.5
*Diarrhoea*	0.044 [33]	5.5
*Dyspepsia*	0.086 [33]	12.0
*Nausea*	0.065 [33]	6.0
*Somnolence*	0.085 [33]	0.0
*Headache*	0.115 [33]	2.8
*Sexual dysfunction*	0.049 [33]	44.0
Sleep difficulties	0.08095 [34]	54.0
Discontinuation Symptoms	0.065 [33]	52.0
**Study Medication**	**Expected Utility Decrease due to ADR**^**§**^	**Monthly medication cost**^††^
*Agomelatine*	0.026	60.27
*Venlafaxine*	0.039	18.03
*Escitalopram*	0.035	56.31
*Fluoxetine*	0.026	21.31
*Sertraline*	0.020	17.35
*Generic Venlafaxine*		14.38
*Generic Escitalopram*		45.60
*Generic Fluoxetine*		14.37
*Generic Sertraline*		10.81

^§^ Computed based on frequency of ADR and Utility Decrease assigned to each ADR.

^‡^ Computed based on Experts’ opinion & Government Gazette.

^†^ Computed based on Experts’ opinion & Gross Domestic Product from National Statistics Service.

*Computed based on Experts’ opinion and Drug Price Bulletin.

^††^ Drug Price Bulletin.

##### Estimation of costs

Since the base case analysis was conducted from the societal perspective, both direct and indirect costs were included in the model. However, only the direct health care cost was also considered as sensitivity analysis since the most funding bodies take the health care perspective as their reference case. The direct health care cost encapsulates all the resource consumption incurred for the care of patients during a depressive episode or remission. In particular, costs associated with patient hospitalizations, outpatient visits, medications, laboratory tests and management of adverse events were considered. Data for resource utilization during a depressive episode or remission were based on expert opinion and reflect the local practice (Additional file 2). The number of utilized resources was combined with the corresponding unit cost, obtained from Government Gazzette or published studies, in order to calculate the total direct cost for each health state. These direct costs were assigned to the relative health states in case of all involved comparators with the exception of agomelatine. In patients under agomelatine treatment, an additional direct cost related to hepatic control recommended was considered. In particular, the European Medicines Agency recently (October 2012) suggested blood control at weeks: 3, 6, 12 and 24 [35]. The cost per hepatic test was set at €75.

The management of an adverse event was assumed to require a physician visit and results in drug prescription [33]. Drowsiness or somnolence was not assumed to be treated pharmaceutically. The management of sleep disorder was assumed to require a medical appointment with a physician and a prescription drug, usually in the form of zolpidem (trademarks include Stilnoct®), in the dose of 10 mg once daily. The management of discontinuation symptoms was set to be the same as the management of nausea as this is a frequently occurring symptom following discontinuation from paroxetine [27].

As for the medication cost, the mean daily drug dose was combined with the relevant drug prices (calculated as the cost per mg). Drug prices were obtained from the drug price bulletin issued by the Greek Ministry of Health in April 2012 [36]. Two main scenarios were considered in terms of drug prices. In the first scenario the price of branded drugs was used, while in the second one, the price of generics was used, and this is 60% lower compared to the original. Only the prices were altered in the case of generics and their effectiveness and safety was assumed to be equal to that of the original product. All costs reflect the year 2012.

Indirect cost consists of productivity loss due to morbidity. Mortality was not considered in the present study because of the time frame of the analysis. The data was based on expert opinion and reflects the local setting in Greece. The cost borne by a missed day of work was calculated on the basis of the average gross domestic product per capita (20,696€) on grounds of the latest data obtained from the National Statistics Service (http://www.statistics.gr) divided with 300 (i.e. the number of assumed working days per year). Table 2 presents information of the total cost per cycle for the different health states (direct and indirect cost for depressive episode and remission, separately), as well as the cost of anti-depressant medication, and that of managing adverse events, sleep disorders and discontinuation symptoms.

### Analysis

The cost-effectiveness of agomelatine relative to its comparators was evaluated with estimation of its incremental cost-effectiveness ratio (ICER), that is its incremental cost per quality-adjusted life year (QALY) saved. Following the literature, when agomelatine was more effective (i.e. higher QALY) and less costly than its comparators, it was considered a “dominant” treatment. In case where agomelatine was more effective and more costly it was assessed whether the ICER was lower than specific predetermined thresholds, including €40,000/QALY to €50,000/QALY which are commonly used in other countries or €60,000/QALY which corresponds to the three times the per capita Gross Domestic Product of Greece, as recommended by the World Health Organisation [37-39].

To identify key model parameters and their impact on the results, one-way deterministic sensitivity analyses using extreme values for all model parameters was used. However, the majority of input data used in the current model are subjected to variation. Therefore, in order to deal with uncertainty, probabilistic sensitivity analysis was performed using a second-order Monte Carlo simulation. In this analysis, a probability distribution was assigned in each parameter (i.e. costs, transition probabilities, utilities etc) and the cost-effectiveness results were recomputed after selecting simultaneously at random values from those distributions. Distributions were selected based on the nature of the variables considered [14]. In particular, probabilities and utility values were constricted on the interval zero to one and hence they are varied according to a beta distribution. For relative risks and costs, the logarithms were assumed to be normally distributed (i.e. log-normal distribution). In general, the distribution parameters (Additional file 3) were estimated based on mean and standard deviations of published data (if available), while if no information on the variability of some parameters was available, their standard deviation was assumed to be equal to 10% of the mean [40]. Then, 1,000 estimates of costs, QALYs, and incremental cost per QALY saved were obtained by applying the bootstrapping technique. A cost-effectiveness acceptability curve was also plotted, and shows the proportion of simulations that are considered cost-effective at different thresholds of willingness to pay (WTP) per gained unit of QALY [41]. All statistical calculations and computations were performed using Microsoft Excel 2007.

## Results

The base case analysis showed that the average total cost of agomelatine-treated patients is lower compared to branded escitalopram, fluoxetine, sertraline and generic fluoxetine and sertraline, while it is slightly higher compared to branded venlafaxine, generic venlafaxine and escitalopram (€14, €37 and €48, respectively) (Table 3). Moreover, Markov model predicted that the average number of QALYs for agomelatine-treated patients is higher compared to all of its comparators. In particular, the increment in QALYs ranged between 0.015 relative to escitalopram to 0.034 relative to sertaline. Therefore, agomelatine is dominant compared to all branded comparators with the exception of venlafaxine, and compared to generic fluoxetine and sertraline. Moreover, it seems to be cost-effective compared to branded venlafaxine (ICER: €547/QALY), generic venlafaxine (ICER: €1,446/QALY gained) and generic escitalopram (ICER: €3,303/QALY gained).

**Table 3:** Base case results of cost-effectiveness analysis (both direct and indirect costs were considered in the analysis)

**Direct comparison**	**Total cost**	**QALYs**	**Incremental cost**	**Incremental QALYs**	**ICER**
			**Agomelatine vs. comparator**	**Agomelatine vs. comparator**	
Agomelatine	€5,434	1.461	-	-	-
Venlafaxine	€5,420	1.436	€14	0.026	€547/QALY
Sertraline	€5,650	1.427	- €215	0.034	Agom. dominant
Escitalopram	€5,462	1.447	- €28	0.015	Agom. dominant
Fluoxetine	€5,563	1.431	- €129	0.030	Agom. dominant
Generic Venlafaxine	€5,397	1.436	€37	0.026	€1,446/QALY
Generic Sertraline	€5,600	1.427	-€166	0.034	Agom. dominant
Generic Escitalopram	€5,386	1.447	€48	0.015	€3,303/QALY
Generic Fluoxetine	€5,508	1.431	-€74	0.030	Agom. dominant

*QALY* Quality Adjusted Life Year.

*ICER* Incremental cost effectiveness ratio.

**Figure 2:**
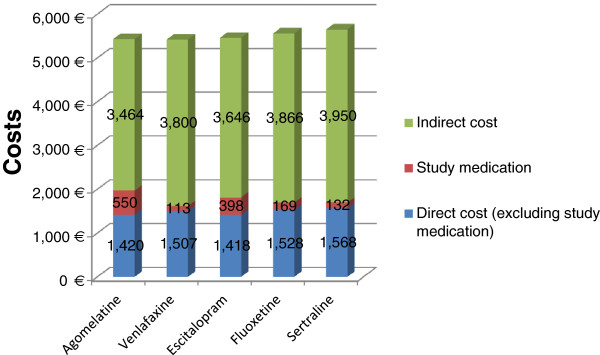
Cost components for agomelatine, venlafaxine, escitalopram, fluoxetine, sertraline.

Figure 2 illustrates the decomposition of total cost for all branded comparators. It was found that treatment of MDD with agomelatine is associated with slightly higher medication cost and lower indirect cost compared to all comparators (branded and generics). In particular, the model used reveals that the medication cost ranges between €550 and €113 for agomelatine and branded venlafaxine, respectively. Finally, it is clear that the total cost related to anti-depressant treatment is mainly driven by the indirect cost for all comparators.

When the indirect cost was excluded from the analysis, agomelatine remains a cost-effective alternative over all branded and generic comparators at the predetermined WTP threshold of €50,000 per QALY gained (Table 4). Further, one-way sensitivity analyses revealed that agomelatine remains dominant against sertraline, fluoxetine, and escitalopram and cost-effective against venlafaxine when ADR, sleep disorders and discontinuation are excluded from the analysis, or when relapse risk, recurrence, and suicide risk during episode are varied. Agomelatine is no longer dominant but remains cost-effective, given established WTP thresholds, when the time horizon is reduced to one year. On the other hand, when the monthly remission rate is increased dramatically for venlafaxine (50.3%) and escitalopram (54.4%), agomelatine is dominated by the comparators.

**Table 4:** Results of cost-effectiveness analysis when only direct health care costs were considered in the analysis

**Direct comparison**	**Total cost**	**Incremental cost**	**ICER**
		**Agomelatine vs. comparator**	
Agomelatine	€1,970	-	-
Venlafaxine	€1,620	€350	€13,682/QALY
Sertraline	€1,701	€269	€7,959/QALY
Escitalopram	€1,816	€154	€10,591/QALY
Fluoxetine	€1,697	€273	€9,183/QALY
Generic Venlafaxine	€1,597	€373	€14,581/QALY
Generic Sertraline	€1,651	€319	€9,434/QALY
Generic Escitalopram	€1,740	€230	€15,799/QALY
Generic Fluoxetine	€1,642	€328	€11,027/QALY

*QALY*: Quality Adjusted Life Year.

*ICER*: Incremental cost effectiveness ratio.

**Figure 3:**
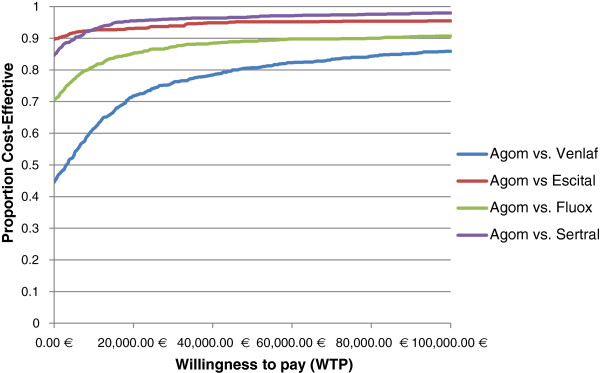
Probability that agomelatine is cost-effective against branded comparators at alternative monetary values of a quality-adjusted-life-year, when both direct health care costs and indirect costs were considered in the analysis.

Finally, the acceptability curve indicates that agomelatine is dominant in 44.5%, 89.6%, 70.6% and 84.6% of simulated samples against branded venlafaxine, escitalopram, fluoxetine and sertraline, respectively. Moreover, agomelatine was found to be cost-effective compared to branded venlafaxine, escitalopram, fluoxetine and sertraline in 80.7%, 95.2%, 89.1% and 96.8%, respectively, at a WTP threshold of €50,000/QALY gained (Figure 3).

Further probabilistic sensitivity analysis revealed similar results when the price of generic comparators was considered in the model. In particular, Agomelatine was found to be cost-effective at a WTP threshold of €50,000/QALY gained in 80.4%, 94.8%, 88.8% and 96.6% of simulated samples, compared to generic venlafaxine, escitalopram, fluoxetine and sertraline, respectively.

**Figure 4:**
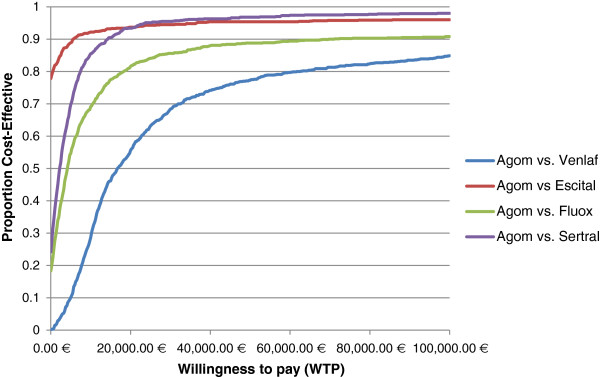
Probability that agomelatine is cost-effective against branded comparators at alternative monetary values of a quality-adjusted-life-year, when only direct health care costs were considered in the analysis.

In case that only direct health care costs were considered in the analysis, the acceptability curve indicates that agomelatine is dominant in 0.2%, 77.8%, 18.3% and 24.8% of simulated samples against branded venlafaxine, escitalopram, fluoxetine and sertraline, respectively. Moreover, agomelatine was found to be cost-effective compared to branded venlafaxine, escitalopram, fluoxetine and sertraline in 77.3%, 90.2%, 88.1% and 90.8%, respectively, at a WTP threshold of €50,000/QALY gained (Figure 4).

## Discussion

In the present study, a Markov model was adapted for the health economic evaluation of agomelatine in MDD against venlafaxine, sertraline, fluoxetine, and escitalopram. The parameters of the Markov model are based on clinical data, published estimates from the literature and expert opinion. The main economic outcome considered is the societal cost and the main effectiveness measure is the QALYs in each option. This is the first study comparing agomelatine with SSRIs and SNRIs in terms of cost-effectiveness. According to the results of the present study, agomelatine dominates all active comparators (i.e. is cost saving for the payer and in parallel improves effectiveness) except for venlafaxine, where agomelatine was found to provide greater health benefit with a slightly higher cost, which equals €547 per QALY gained. These results remain stable when generic products are considered in the model, with the exception being the case of generic escitalopram where agomelatine was found to be cost-effective instead of dominant. These results are confirmed in the vast majority of scenarios examined in the one-way sensitivity analysis. Only in the case that the monthly remission rate is increased dramatically for venlafaxine (50.3%) and escitalopram (54.4%), agomelatine is dominated by the comparators. However, these remission rates are based on the CGI scale-in the case of venlafaxine - and on a longer observational period - 12 weeks instead of 6 and 8 weeks in the case of escitalopram – and may not at all be comparable to remission rates based on HAM-D or MADRS. Major drivers of the results are the rates of remission, relapse and discontinuation, as well as the cost of drugs. Of these, the remission rates are the single most important driver of incremental costs and effects. ADRs only influence results to a lesser extent, due to their relatively low cost and utility decrement compared to the different depression states.

The current model combines data from several different sources: expert opinion, published clinical trial results, patient-level data for some trials, published longitudinal data on costs and utilities in depression, as well as other published data for baseline risks and the effect of side-effects. A strength of the model is that it includes effectiveness data, as well as sleep disorder and relevant side effects from clinical trials. The model also allows for extensive sensitivity analyses to test the robustness of the cost-effectiveness results. The analysis pursued is characterized by specific drawbacks and limitations. First of all, limitations in the model arise from the nature of the underlying data, which in several cases were not available with the required level of detail. We have sought to balance this by using conservative assumptions where possible, such as assuming the same clinical efficacy as for agomelatine where no relevant data was available. Moreover, the results have to be considered in the strict Greek setting and on the basis of the present time resource and drug prices. If any of the underlying parameters change, so may the results and the conclusions of the analysis.

At this point, it should be noticed that the results of such a pharmacoeconomic study should be considered in conjunction with a list of other factors to make a decision on antidepressant treatment. Based on a recently published review by Himmerich & Wranik, the potential determinants of antidepressant treatment choice are classified into seven categories, including illness and treatment characteristics, patient and physician characteristics, treatment setting characteristics, decision supports and pharmacoeconomic aspects [42].

## Conclusion

In conclusion, clinical data based on a head-to-head comparison of agomelatine with venlafaxine, sertraline fluoxetine or escitalopram were used together with local resource utilization and price data, to evaluate whether agomelatine is a cost-effective for the treatment of major depressive disorder in the Greek setting from a societal perspective. The present economic evaluation indicates that agomelatine provides greater benefit and is less costly compared to escitalopram, generic fluoxetine and generic sertraline and it may be cost-effective compared to generic venlafaxine. Thus it provides a good value for money option for the management of the group of patients and the setting of the evaluation.

## Competing interests

NM received unrestricted grant from Servier- Hellas. However, the study sponsor had no interference in the study design, data collection or writing of the manuscript. None of the rest of authors has any personal or financial competing interest.

## Authors’ contributions

GK adapted the model, conducted the analyses, interpreted the results and wrote the manuscript. NM supervised the study, contributed to results interpretation, and reviewed the manuscript. IC and TM were the medical experts provided local input data and reviewed the manuscript. LJ developed the model and reviewed the manuscript. All authors read and approved the final manuscript.

## Pre-publication history

The pre-publication history for this paper can be accessed here:

http://www.biomedcentral.com/1472-6963/13/173/prepub

## Supplementary Material

Additional file 1 Relapse survival curve. Click here for file

Additional file 2 Resource utilization (excluding management of adverse events and antidepressants) and unit costs. Click here for file

Additional file 3 Distributions and model parameters used in the stochastic analysis. Click here for file
