# Supplementary material for: Economic evaluation of agomelatine relative to other antidepressants for treatment of major depressive disorders in Greece
Source: BMC Health Serv Res. 2013 May 10;13:173. doi: 10.1186/1472-6963-13-173 (PMC3654993; doi:10.1186/1472-6963-13-173)
Supplement: Additional file 1 — Relapse survival curve. [file 1472-6963-13-173-S1.doc]

**Additional file 1**

| Relapse survival curve | | | | |
| --- | --- | --- | --- | --- |
|  | **Estimates** | | **Standard error** | |
|  | **Shape (α)** | **Constant (β)** | **Shape (α)** | **Constant (β)** |
| **Agomelatine 25/50 mg** | 0.789 | -5.434 | 0.128 | 0.665 |
| **Placebo** | 1.007 | -5.730 | 0.109 | 0.555 |
