# Supplementary material for: Economic evaluation of agomelatine relative to other antidepressants for treatment of major depressive disorders in Greece
Source: BMC Health Serv Res. 2013 May 10;13:173. doi: 10.1186/1472-6963-13-173 (PMC3654993; doi:10.1186/1472-6963-13-173)
Supplement: Additional file 2 — Resource utilization (excluding management of adverse events and antidepressants) and unit costs. [file 1472-6963-13-173-S2.doc]

**Additional file 2**

| Resource utilization (excluding management of adverse events and antidepressants) and unit costs. | | | | | |
| --- | --- | --- | --- | --- | --- |
|  | **Depressive Episode (6 months)** | | **Remission (6 months)** | |  |
| **Indirect cost** | **Percent of utilization** | **Length** | **Percent of utilization** | **Length** | **Unit cost** |
| Productivity loss | 100% | 22 days | 100% | 15 days | €69/day |
| Care giver | 50% | 22 days |  |  |  |
| **Direct Cost** | **Percent of utilization** | **Length** | **Percent of utilization** | **Length** |  |
| Hospitalization | 6.5% | 35 | - | - | €320/day |
| Outpatient Visits  *Psychiatrists*  *Other Health professionals* | 100%  100% | 7  1 | 100%  100% | 3  1 | €50/visit |
| Medication other than  *Anxiolytics* | 100% | 40 | - | - | €0.04/day |
| Antidepressants |  |  |  |  |  |
| *Agomelatine* |  |  |  |  | €1.98/day |
| *Venlafaxine*  *Branded*  *Generic* |  |  |  |  | €0.59/day  €0.47/day |
| *Sertraline*  *Branded*  *Generic* |  |  |  |  | €0.57/day  €0.36/day |
| *Escitalopram*  *Branded*  *Generic* |  |  |  |  | €1.85/day  €1.50/day |
| *Fluoxetine*  *Branded*  *Generic* |  |  |  |  | €0.70/day  €0.47/day |
