# Supplementary material for: Economic evaluation of agomelatine relative to other antidepressants for treatment of major depressive disorders in Greece
Source: BMC Health Serv Res. 2013 May 10;13:173. doi: 10.1186/1472-6963-13-173 (PMC3654993; doi:10.1186/1472-6963-13-173)
Supplement: Additional file 3 — Distributions and model parameters used in the stochastic analysis. [file 1472-6963-13-173-S3.doc]

**Additional file 3**

| Distributions and model parameters used in the stochastic analysis | | | |
| --- | --- | --- | --- |
| **Utility** | **Beta distribution** | | **Sources** |
|  | **α** | **β** | Sobocki et al. (2006)[17] |
| Healthy | 1277.5 | 208.0 |  |
| Remission | 531.0 | 124.6 |  |
| Depressive episode | 264.0 | 199.2 |  |
|  |  |  |  |
|  | **Log-normal distribution** | |  |
|  | **μ** | **σ** |  |
| **Direct costs (excluding medication)** |  |  | Computed by investigators |
| Remission | 3.555 | 0.116 |  |
| Depressive episode | 5.247 | 0.101 |  |
| **Indirect costs** |  |  | Computed by investigators |
| Remission | 5.153 | 0.099 |  |
| Depressive episode | 5.940 | 0.101 |  |
|  |  |  |  |
| **Transition probabilities** |  |  |  |
| Relapse |  |  |  |
| *Agomelatine and venlafaxine* | Bootstrapping | | Rouillon et al. (2007)[27] |
| *Placebo* | Bootstrapping | | Rouillon et al. (2007)[27] |
|  |  |  |  |
|  | **Normal distribution (of logarithm, shown for SD)** | |  |
|  | **μ** | **σ** |  |
| *Escitalopram (relative risk)* | 0.531 | 0.2199 | Meta-analysis vs placebo |
| *Fluoxetine (relative risk)* | 0.513 | 0.1355 | Meta-analysis vs placebo |
| *Sertraline (relative risk)* | 0.633 | 0.3047 | Meta-analysis vs placebo |
|  |  |  |  |
| Remission |  |  |  |
|  | **Beta distribution** | |  |
|  | **α** | **β** |  |
| *Agomelatine* | 128.02 | 268.32 | Meta-analysis of studies of Hale et al. and Kasper et al. [22,21] |
| *Venlafaxine* | 128.02 | 268.32 | Assumed equal to Agomelatine |
| *Escitalopram* | 128.02 | 268.32 | Assumed equal to Agomelatine |
| *Fluoxetine* | 72.70 | 183.30 | Hale et al.[21] |
| *Sertraline* | 44.72 | 110.28 | Kasper et al.[22] |
|  |  |  |  |
|  | **μ** | **σ** |  |
| Mortality multiple, depressive episode | 20.35 | 1.1 | Harris et al (1997)[28] |
|  |  |  |  |
| **Sleep difficulties** |  |  |  |
|  | **Beta distribution** | |  |
|  | **α** | **β** |  |
| Probability of sleep difficulties during depressive episode |  |  |  |
| *Agomelatine* | 4.18 | 592.32 | Meta-analysis of studies[20-23] |
| *Venlafaxine* | 3.97 | 163.03 | Lemoine et al. (2007)[20] |
| *Escitalopram* | 3.98 | 155.03 | Quera-Salva (2011)[23] |
| *Fluoxetine* | 4.98 | 257.02 | Hale et al.(2010)[21] |
| *Sertraline* | 2.99 | 155.01 | Kasper et al. (2010)[22] |
|  |  |  |  |
| Utility decrease due to sleep difficulties | 1.886 | 21.408 | Botteman et al. (2007)[32] |
|  |  |  |  |
|  |  |  |  |
| **Adverse drug reactions (ADR)** | **Beta distribution** | |  |
| Utility loss | **α** | **β** |  |
| *Constipation* | 55.83 | 803.14 | Sullivan et al (2004)[31] |
| *Diarrhea* | 58.33 | 1267.26 |  |
| *Dyspepsia* | 53.58 | 569.39 |  |
| *Nausea* | 55.83 | 803.14 |  |
| *Somnolence* | 57.52 | 619.23 |  |
| *Headache* | 55.45 | 426.75 |  |
| *Sexual dysfunction* | 56.21 | 1090.84 |  |
|  |  |  |  |
| *Probability distributions for ADR - Agomelatine* |  |  | Meta-analysis of studies[20-23] |
| Constipation | 19.86 | 715.63 |  |
| Diarrhea | 29.46 | 706.96 |  |
| Dyspepsia | 11.64 | 570.41 |  |
| Nausea | 48.21 | 682.20 |  |
| Somnolence | 25.90 | 714.09 |  |
| Headache | 81.54 | 653.05 |  |
| Sexual dysfunction | 2.82 | 561.99 |  |
|  |  |  |  |
| *Probability distributions for ADR - venlafaxine* |  |  | Lemoine et al. (2007)[20] |
| Constipation | 6.96 | 160.04 |  |
| Diarrhea | 2.99 | 164.01 |  |
| Dyspepsia | 4.01 | 162.99 |  |
| Nausea | 37.78 | 129.22 |  |
| Somnolence | 7.95 | 159.05 |  |
| Headache | 19.87 | 147.13 |  |
| Sexual dysfunction | 29.893 | 1.640.107 |  |
|  |  |  |  |
| *Probability distributions for ADR - escitalopram* |  |  | Quera-Salva (2011)[23] |
| Constipation | 0.99 | 158.01 |  |
| Diarrhea | 10.94 | 148.06 |  |
| Dyspepsia | 3.98 | 155.03 |  |
| Nausea | 21.86 | 137.14 |  |
| Somnolence | 5.96 | 153.04 |  |
| Headache | 22.86 | 136.14 |  |
| Sexual dysfunction | 1.99 | 157.01 |  |
|  |  |  |  |
| *Probability distributions for ADR - fluoxetine* |  |  | Hale et al.(2010) [21] |
| Constipation | 2.99 | 259.01 |  |
| Diarrhea | 6.97 | 255.03 |  |
| Dyspepsia | 1.99 | 260.01 |  |
| Nausea | 29.89 | 232.11 |  |
| Somnolence | 8.96 | 253.04 |  |
| Headache | 29.89 | 232.11 |  |
| Sexual dysfunction | 1.00 | 261.00 |  |
|  |  |  |  |
| *Probability distributions for ADR - sertraline* |  |  | Kasper et al. (2010)[22] |
| Constipation | 0.99 | 157.01 |  |
| Diarrhea | 8.94 | 149.06 |  |
| Dyspepsia | 1.99 | 156.01 |  |
| Nausea | 6.95 | 151.05 |  |
| Somnolence | 1.99 | 156.01 |  |
| Headache | 15.89 | 142.11 |  |
| Sexual dysfunction | 2.99 | 155.01 |  |
|  |  |  |  |
| **Discontinuation of active treatment** |  |  |  |
|  | **Beta distribution** | |  |
|  | **α** | **β** |  |
| Agomelatine | 86.02 | 649.21 | Meta-analysis of studies[20-23] |
| Venlafaxine | 35.78 | 130.22 | Lemoine et al. (2007)[20] |
| Escitalopram | 1.112 | 0.1835 | Quera-Salva (2011)[23] |
| Fluoxetine | 1.420 | 0.1052 | Hale et al (2010)[21] |
| Sertraline | 1.478 | 0.1294 | Kasper et al.(2010) [22] |
|  |  |  |  |
| **Discontinuation symptom** |  |  |  |
|  | **Beta distribution** | |  |
|  | **α** | **β** |  |
| *Probability of discontinuation symptoms* |  |  |  |
| Agomelatine | - | - | Montgomery et al. (2004)[24] |
| Venlafaxine | 8.40 | 33.60 | Montgomery et al. (2004)[24] |
| Escitalopram | 2.94 | 39.06 | Montgomery et al. (2004)[24] |
| Fluoxetine | n/a | n/a | No data available |
| Sertraline | n/a | n/a | No data available |
